# Antidiabetic potential of two medicinal plants used in Gabonese folk medicine

**DOI:** 10.1186/s12906-016-1052-x

**Published:** 2016-02-22

**Authors:** Huguette Agnaniet, Elvis Jolinom Mbot, Ousmane Keita, Jean-Alain Fehrentz, Anita Ankli, Audrey Gallud, Marcel Garcia, Magali Gary-Bobo, Jacques Lebibi, Thierry Cresteil, Chantal Menut

**Affiliations:** Laboratoire de Substances Naturelles et de Synthèses Organométalliques (LASNSOM), Université des Sciences et Techniques de Masuku, Faculté des Sciences B.P. 943, Franceville, Gabon; Institut des Sciences Appliquées (ISA)-Département de Génie Biologique (GB)-Biochimie-Université des Sciences, des Techniques et des Technologies de Bamako (USTTB) BPE, 423 Bamako - Hamdallaye ACI 2000 - Rue : 405, Porte, 359 Mali; Institut des Biomolécules Max Mousseron (IBMM) UMR 5247 CNRS-Université Montpellier-ENSCM, Bâtiment E, Faculté de Pharmacie, 15, avenue Charles Flahault BP14491, 34093 Montpellier, cedex 5 France; CAMAG Laboratory, Sonnenmattstrasse 11, 4132 Muttenz, Switzerland; CIBLOT, IPSIT - IFR141, 5 rue Jean Baptiste Clément, 92290 Chatenay-Malabry, France

**Keywords:** *Sarcocephalus pobeguinii*, *Nauclea diderrichii*, α-, β-glucosidases inhibition, Antidiabetic activity

## Abstract

**Background:**

Diabetes mellitus is a metabolic disorder which is rising globally in rich and developing countries. In the African region this rate is the highest, with 20 million diagnosed diabetics. Despite a noticeable progress in the treatment of diabetes mellitus by synthetic drugs, the search for new natural anti-diabetic agents is going on. *Nauclea diderrichii* (De Wild.) Merr. (*ND*) and *Sarcocephalus pobeguinii* Hua ex Pellegr. (*SP*) are used as traditional medicines in Gabon for the treatment of different diseases, especially in the case of diabetes. The aim of this study was to evaluate the antidiabetic potential of these two medicinal plants traditionally used in Gabon.

**Methods:**

Pharmacological (inhibitory action on α and β-glucosidases) and toxicological (effect on human T cell proliferation) studies were conducted on aqueous extracts of *ND* (leaves and bark) and *SP* (bark) collected in Gabon. All raw extracts were analyzed by HPTLC and their content in phenolic compounds was determined by using standard method. The most active extracts were submitted to preparative HPLC in order to evidence the most efficient subfractions by biological evaluation.

**Results:**

The results showed that two extracts from *ND* were potent α-glucosidase inhibitors, the leaf extract being more active that the bark extract: the first one was more than 60 fold more active than Acarbose, which is an oral medication used to treat type 2 diabetes; the extract from *SP* bark was less efficient. The HPLC subfractions of the extracts of *ND* leaves and *SP* bark were tested in the same experimental conditions. In each case, the most active subfractions still show very potent inhibitory effect on α-glucosidase (80-90 % inhibition at 0.1 mg/mL). The most efficient extract, from *ND* leaves, was also characterized by the highest percentage of phenolic compounds, which suggests a relationship between its inhibitory potential on α-glucosidase and its content in phenolic compounds. Conversely, only a moderate inhibitory activity of the three extracts was observed on β-glucosidase.

**Conclusion:**

These results clearly indicated that active compounds present in *N. diderrichii* and *S. pobeguinii* leaves or/and bark were selective and highly potent inhibitors of α-glucosidase and validate their popular use for the treatment of diabetes.

**Electronic supplementary material:**

The online version of this article (doi:10.1186/s12906-016-1052-x) contains supplementary material, which is available to authorized users.

## Background

Diabetes mellitus is a metabolic disorder, mainly of two types (1 and 2) and characterized by chronic hyperglycemia with disturbances of carbohydrate, fat and protein metabolism resulting from defects in insulin secretion, insulin action or both [1–3]. Currently, the number of diabetics sharply increases in rich and developing countries. In 2013, the overall prevalence of diabetes in the world’s population was estimated to 8.3 % approximately [4]. In the African region, and particularly in Sub-Saharan Africa, this rate is the highest, with 20 million diagnosed diabetics [5].

Oral hypoglycemic agents, such as α-glucosidase inhibitors, exert their effects *via* a variety of mechanisms, which include the reduction of hepatic glucose production, the enhancement of insulin secretion by pancreatic β-cells and the improvement of insulin sensitivity. Glucosidase inhibitors have been the subject of extensive interest [6] because of their potential as drugs for the treatment of diabetes [7]. Alpha-glucosidase is a membrane-bound enzyme, at the epithelium of the small intestine; it hydrolyses the cleavage of glucose from disaccharides and oligosaccharides. The inhibitors of this enzyme delay carbohydrate hydrolysis, prolong the overall carbohydrate digestion time and thus cause a reduction in glycaemia [8]. Therefore, inhibition of α-glucosidase is considered as an important way to treat non-insulin-dependent diabetes. This hypothesis was successfully confirmed in animal and clinical studies by the administration of various α-glucosidase inhibitors [9, 10].

Despite considerable progress in the treatment of diabetes mellitus by synthetic drugs, the search for new natural anti-diabetic agents is going on. The world health organization (WHO) estimates that 80 % of the population in some developing countries, especially in Sub-Saharan Africa, still use the traditional medicine [11]. Several plants are used in folk medicine for their hypoglycemic activity and they were investigated using different experimental methods [12]. Thanks to these studies, natural glycosidase inhibitors have been discovered and tested for their activity [13, 14].

Gabon has remarkable biodiversity and rich cultural traditions of plant use. Scientific understanding of medicinal plants is however largely unexplored and pharmacological investigation of Gabonese flora only gained momentum recently. Rubiaceae are among plants of wide usage in traditional medicine in the Sub-Saharan region [15] more particularly *Nauclea* and *Sarcocephalus* species, which belong to the tribe Naucleae, subtribe Naucleinae [16]. In Gabon, the species *Nauclea diderrichii* and *Sarcocephalus pobeguinii* (syn. *Nauclea pobeguinii*) are used in folk medicine for the treatment of different diseases, especially in the case of diabetes associated or not to hypertension. In the Eviya area, *S. pobeguinii* is also used in leaf infusions as febrifuge, while bark maceration is indicated for urogenital infections [17]. The traditional use of both species is also mentioned in ethnomedicinal reports from other African countries: *N. diderrichii* is used in Nigeria against arthritis and malaria [18, 19] while *S. pobeguinii* was reported for several indications in Cameroon [20], Nigeria [21, 22], Guinea [23] and Senegal [24].

*N. diderrichii* is an evergreen tree that reaches a height of 30-40 m and a diameter of 0.9-1.5 m; its natural habitat is subtropical or tropical moist lowland forests; the wood of this tree, which is known as bilinga, is dense and resistant to fungi and insects [25]. *S. pobeguinii* is a forest tree or shrubby tree 6–30 m tall; its bark is whitish, grey or pale brown, roughly fissured, flaking in papery scales about 2 cm in diameter; its fruits are edible [26].

The chemical investigations on these two species are numerous: they concern alkaloids from *N. pobeguinii* [27, 28] whereas alkaloids [29–31], saponins [32, 33] and other chemical classes [34, 35] were obtained from different plant parts of *N. diderrichii*. A few biological studies have been conducted on these species. Water or/and ether extracts of *N. diderrichii* from Gabon were evaluated for their anti-malarial [36] and anti-leshmanial [37] activities. The same species collected in the West African region was screened for its antiplasmodial activity [38].

Finally, ethanol extracts from *N. pobeguinii* bark were also tested for their antimalarial activity [39–42].

Nevertheless, to the best of our knowledge, *N.diderrichii* and *S. pobeguinii* were not screened for their hypoglycemic activity despite their traditional use for diabetes. A literature survey shows only some studies in this field on plants belonging to the same genera, such as *Sarcocephalus latifolius* from the Central Region of Togo [43] and Nigeria [44] or *Nauclea latifolia* collected in Benin (roots and stem) [45] or in Nigeria (leaves) [46]. This led us to carry out phytochemical and biological investigations on the two species collected in Gabon: bark and leaf extracts of *N. diderrichii* as well as bark extract of *S. pobeguinii*.

Plant extracts were prepared and tested in human cells to determine their capacity to inhibit α- and β-glucosidases, to investigate their biocompatibility with cells and to provide pharmacological basis for their traditional use as anti-diabetic agent.

## Methods

### Plants materials

The plant selection was based on their use by local population and traditional healers. Thirty people were interviewed in 10 villages located in the Nkomo Mondah department (Province de l’Estuaire), in the Ogooué as well as in the Lakes departments (Province du Moyen Ogooué). These data were supplemented by literature information [17, 23, 47–49]. The plants were collected during the short dry season: *N. diderrichii* was collected in Libreville in December 2009 (department of Nkomo-Mondah) and *S. pobeguinii* was collected in Lambarene in January 2010 (department of Ogooue). They were identified by Y. Issembe and R. Niangadouma, botanists at the National Herbarium of the Institute of Pharmacopea and Traditional Medicine (IPHAMETRA) where voucher specimens were deposited under the following numbers: *Nauclea diderrichii* (De Wild.) Merr. [Wilks: 8836 (WAG), 1988; 2438 (LBV, WAG), 1991] and *Sarcocephalus pobeguinii* Hua ex Pellegr. [Azizet Issembé 172 (LBV, WAG), 2000; Wilks: 1035 (LBV, WAG)]. Leaves and bark of *N. diderrichii* as well as bark pieces of *S. pobeguinii* were collected and air dried in a room maintained at a controlled temperature (25 °C) using an air-conditioning unit; then, the samples were stored in a dry place until use.

### Preparation of plants extracts

In the case of *N. diderrichii*, two parts of the plant were extracted: raw leaves and ground bark. Plant material was macerated in water (100 g of plant sample in 1 L of water at room temperature). After filtration on büchner (paper Whatman N°3, medium porosity, particle retention 6 μm, diam. 9 cm), the filtrates were lyophilized to give the samples E 14 (leaves) and E 15 (bark) with 13.9 % yield and 15.0 % yield respectively. The ground bark of *S. pobeguinii* was extracted by decoction. Briefly, 100 g of plant material was boiled in 1 L of water for 30 min. After filtration on paper Whatman N°3 (medium porosity, particle retention 6 μm, diam. 9 cm), the filtrate was cooled at room temperature and lyophilized to obtain 10.34 g of the extract E 10.

### Phytochemical analysis by HPTLC

Phytochemical analysis of the extracts was undertaken by HPTLC method using HPTLC plate silica gel F_254s_ (Merck) 20×10 cm and different derivatizations. All reagents and solvents were of analytic grade.

The samples were tested following the CAMAG Standard Operating Procedures (SOP): 200 mg of dry extracts were dissolved in 10 mL of ethanol:water 1:1, sonicated twice during 10 min and centrifuged. The supernatant was used for the analyses; applications volumes: 2 and/or 5 μL for samples and standard solutions with Automated TLC Sampler 4, band length 8 mm, distance from lower edge 8 mm, distance from left and right edge 20 mm, track distance min. 10 mm; development in the Automatic Developing Chamber ADC2 with chamber saturation during 20 min and with adjustment of relative humidity to 33%rH. The migration distance (from lower edge of plate) was 70 mm; different mobile phases and derivatization reagents were used: S1 (toluene : ethyl acetate 19:1), S2 (chloroform : methanol : water 35:15:2), S3 (ethyl acetate : acetic acid : formic acid : water100:11:11:27), S4 (1-butanol : acetic acid : water 7:1:2) and S5 (chloroform : methanol : water 70:30:4).

The most suitable solvent system combined with the adequate derivatization was selected for the performance: the best resolution was most often observed with mobile phases S2 and S3 for *N. diderrichii* and mobile phase S2 in the case of *S. pobeguinii.*

The HPTLC plates were dipped in the following derivatization reagents:

- Anisaldehyde sulphuric acid reagent (AAS) (for mobile phases S1, S2 and S4) for saponins detection under white light
- NP/PEG reagent (S3) for flavonoids detection under 366 nm
- Dragendorff reagent (S5) for alkaloids detection under white light
- 2,2-Diphenyl-picrylhydrazyl reagent (DPPH) for screening of antioxidant properties of substances (S3).

Observation was performed using TLC Visualizer under UV 254 and 366 nm prior to derivatization and under UV 366 nm and white light illumination (remission and transmission mode) after derivatization.

### Determination of total phenolic compound content

Folin-Ciocalteu reagent F 9252 lot # BCBF 2476 was purchased from Sigma-Aldrich Co (France). Total phenols content was performed by the Folin-Ciocalteu (FC) colorimetry method (FC) [50] which is based on a chemical reduction of the reagent, a mixture of tungsten and molybdenum oxides. Although not very specific for phenolics, the protocol gives a good idea of the total phenols content. Gallic acid was employed as calibration standard and results were expressed as gallic acid equivalents. Briefly, 0.5 mL of gallic acid solution (10-100 mg/L) was mixed with 0.25 mL FC reagent (1 N). After five minutes, 1.25 mL sodium carbonate (20 % w/v) was added. The mixture was shaken and left during one hour at room temperature. Absorbance A was measured at 725 nm with different concentrations (c) of gallic acid. The linear regression A = f(c) was carried out with Microsoft Excel. The same protocol was used with the plants extracts (0.2-1 g/L) to evaluate their phenols content. The results were expressed in mg of gallic acid equivalents (GAE) per g extract. All experiments were repeated three times. The results are expressed as the mean ± standard deviation (SD).

### Fractionation by HPLC

The HPLC purifications were run on a Waters 4000 preparative apparatus on a C18 Deltapak column (100 mm × 40 mm, 15 μm, 100 Å), with UV detection at 214 nm, at a flow rate of 50 mL/min of a mixture of A (water with 0.1 % TFA) and B (acetonitrile with 0.1 % TFA) in a gradient mode. E 10 was purified starting from 100 % A to 100 % B in 20 min after an isocratic run of 5 min in 100 % A. E 14 was purified in a gradient mode from 100 % A to 50 % B in 25 min. The samples were collected manually every two minutes and the fractions were lyophilized.

### α-Glucosidase and β-glucosidase inhibitory assays

Acarbose, α-glucosidase from *Saccharomyces cerevisiae* and β-glucosidase from almond were obtained from Sigma, isofagomine was synthesized as previously reported [51]. Typical procedure for inhibition studies: α- and β-glucosidase activities were assayed with either 8 mM solution of 4-methyl umbelliferyl α-D-glucopyranoside or 4-methyl-umbelliferyl β-D-glucopyranoside (Carbosynth) in 100 mM NaHPO_4_ buffer, pH 6.8 at 30 °C in a 384-well microplate. 10 mM Acarbose and 1 μM isofagomine were used as reference inhibitors for α- and β-glucosidases, respectively. Fluorescence was monitored (λ_excitation_ = 364 nm, λ_emission_ = 445 nm) over a 20 min period, in the presence of the extract/compound solutions in DMSO compared to the same volume of DMSO alone. The fluorescence increase reflected the cleavage of umbelliferyl glucopyranosides: the value 100 % was attributed to the fluorescence obtained with enzymes in the presence of DMSO alone. In incubations with extracts or compounds, remaining activity (RA, expressed as a percentage) was calculated as the ratio [fluorescence released in the presence of extracts/compounds]/[fluorescence released in the presence of DMSO alone] × 100 and shown in Figs. 2, 3, 4 and 5 for every concentration of extract/compound. Finally, the percentage inhibition was calculated from these values following the equation: 100-RA.

For the initial screening, extracts dissolved in DMSO were added at a final concentration of 1 mg/mL. Active extracts and separated fractions were diluted in DMSO and added at concentrations ranging 0.01 to 1 mg/mL in duplicate.

### Cell culture and cytotoxicity assay

Human breast (MCF-7) cancer cells were purchased from ATCC (American Type Culture Collection, Manassas, VA). MCF-7 cells were cultured in DMEM-F12 culture medium supplemented with 10 % foetal bovine serum and gentamycin 50 μg/mL. These cells were allowed to grow in humidified atmosphere at 37 °C under 5 % CO_2_. For cytotoxicity analysis, MCF-7 cells were seeded into 96-well plates at 2.10^4^ cells per well in 200 μL culture medium and allowed to grow for 24 h. Then cells were treated for 48 h with or without extracts at 0.5 and 1 mg/mL. After incubation, a MTT assay was performed to evaluate the cytotoxicity [52]. The assay detects the reduction of MTT [3-(4,5-dimethylthiazolyl)-2,5-diphenyl-tetrazolium bromide] by mitochondrial dehydrogenase to blue formazan products, which reflects the normal functioning of mitochondrial and cell viability [53]. Briefly, 0.5 mg/mL MTT reagent (20 μL) was added to each well and incubated for additional 4 h. Then, the medium was removed and 150 μL of EtOH/DMSO (1:1) was added to MTT precipitates in each well to solubilize the formazan crystals. The plates were read for optical density at 540 nm, using microplates reader (Multiskan).

## Results and discussion

### Ethnobotanical survey

Of all the collected information, it appears firstly that populations refer to the experiences of others to use medicinal plants as a remedy against well-defined diseases in accordance with a transmission of traditional practices from one generation to another, or refer to traditional healers. Usually, the dose remains uncertain. In addition, the traditional healers do not generally identify *stricto sensu* the diabetic or hypertensive pathology, but they treat the characteristic symptoms associated with them. Among all the data, *S. pobeguinii* and *N. diderrichii* were the most cited for the various therapeutic applications mentioned above, and ethnobotanical survey reported the use of macerate or decoction of bark of both species to cure diabetes as well as hypertension. Finally, these species were harvested in their natural habitat.

### Chemical screening

The phenols content of the three extracts obtained from the equation A = 19.392c − 0.0153 (in which A represents the absorbance and c the gallic acid concentration, in g/L) were expressed as gallic acid equivalent (GAE) by gram of extract as follows:

- E 10 = 84.5 mg ± 1.5 GAE/g extract
- E 14 = 148 mg ± 3 GAE/g extract
- E 15 = 75.2 mg ± 2.6 GAE/g extract

Extract 14, obtained from *N. diderrichii* leaves*,* is characterized by the highest percentage of phenolic components while both extracts from bark of *N. diderrichii* (E 15) and *S. pobeguinii* (E 10) contained lower amounts of compounds belonging to this chemical class. These results are corroborated by the HPTLC analysis using DPPH reagent which shows some nice defined white zones for the three extracts, mainly in the case of E14, indicating a high radical scavenging effect, mainly located in the polar fraction (see Additional file 1).

The other HPTLC experiments using different derivatization reagents (HPTLC plates not shown) revealed the presence of flavonoids, alkaloids and saponosids in the extracts (Table 1). These results confirm the literature data which report the presence of alkaloids, flavonoids, steroids and glycosides [27, 28] in *S. pobeguinii*. The isolation of alkaloids and saponins (quinovic acid glycosides) from *N. diderrichii* has also been reported [29–34].

**Table 1:** Phytochemical test results by HPTLC of *S. pobeguinii* and *N. diderrichii* extracts

Extract	Alkaloid	Saponin	Flavonoid	Radical scavenging activity
*S. pobeguinii* bark(E10)	+	++	+	+
*N. diderrichii* leaf (E14)	+	+	++	++
*N. diderrichii* bark (E15)	+	+	++	+

Sign (+) indicates present (++ for more intense zones) and sign (-) indicates absent

### Cytotoxicity evaluation

**Fig. 1:**
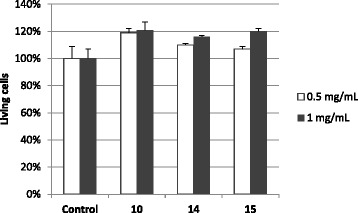
Cytotoxicity of extracts 10, 14 and 15 on the human cell line MCF-7. Cells were incubated or not (Control) with 0.5 or 1 mg/mL of each extract for 48 h. Cell viability was quantified by MTT assay. Data are mean ± SD of 2 independent experiments

Finally, in order to verify the safety of these extracts, cytotoxicity experiments were performed on human MCF-7 cell line. Cells were incubated 48 h with the extracts 10, 14 and 15 previously dissolved in phosphate buffer, at final concentrations of 0.5 and 1 mg/mL. Cell viability results are represented in Fig. 1. Compared to the control incubated with phosphate buffer alone, there is no decrease in the number of living cells after treatment with E 10, E 14 and E 15. Data show that, in the conditions used in this study, no cytotoxicity was observed on human cells treated with the extracts 10, 14 and 15. However, this is only preliminary experiments performed to verify the absence of intrinsic toxicity of the products studied. Data need to be reproduced on pancreatic cells.

### Preliminary screening of raw extracts on α-glucosidase activity

**Fig. 2:**
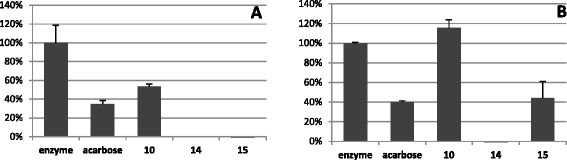
Inhibition of α-glucosidase by extracts 10, 14 and 15. α-Glucosidase was incubated with extracts at a concentration of 1 (panel **a**) and 0.1 mg/mL (panel **b**). Remaining activities in the presence of extracts are expressed as a percentage of enzyme activity incubated in DMSO alone and are the mean ± SE of three separate determinations. 10 mM Acarbose was used as specific inhibitor of α-glucosidase

The two extracts from *N. diderrichii* (E 14 and E 15) and the one from *S. pobeguinii* (E 10) were tested for their capacity to inhibit α-glucosidase. The extracts from N. *diderricchii* were very potent α-glucosidase inhibitors and totally impede the enzymatic activity at 1 mg/mL concentration (Fig. 2a). Both still retained a high activity at 0.1 mg/mL (Fig. 2b), the leaf extract (E 14) being more active than the bark extract (E 15). Tested in the same conditions, extract 10 isolated from *S. pobeguinii* was less active (45 and 0 % inhibition at 1 and 0.1 mg/mL). Finally, the inhibiting capacity of the most active samples (E14 and E 15) was severely reduced at 0.01 mg/mL (see Additional file 2).

Nevertheless, it is noteworthy that the extracts from *N. diderrichii* (E 14 and E 15) displayed a higher inhibitory potency than the reference compound acarbose, which is an oral medication used to treat type 2 diabetes. Indeed, E14, which induced 100 % inhibition at 0.1 mg/mL, was more than 60 fold more active than Acarbose (63 % inhibition at 10 mM final concentration = 6.45 mg/mL).

The high activity observed for the extract obtained from of *N. diderrichii* leaves (E 14), which is the richest in phenolic components, suggests a direct relationship between its inhibitory potential of α-glucosidase and its content in phenolic compounds.

### Selective inhibition by raw extracts

**Fig. 3:**
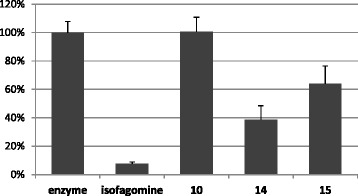
Inhibition of β-glucosidase by extracts 10, 14 and 15. β-Glucosidase was incubated with extracts at a concentration of 1 mg/mL. Remaining activities in the presence of extracts are expressed as a percentage of enzyme activity incubated in DMSO alone and are the mean ± SE of three separate determinations. 1 μM Isofagomine was used as specific inhibitor of β-glucosidase

We further evaluated the inhibitory potential of raw extracts on the β-hydrolysis of glucopyranosides to ascertain the stereospecificity of the active compounds. Evaluation of inhibitory efficiency of the three extracts on β-glucosidase indicated a moderate activity of E 14 and E 15 at 1 mg/mL (with a maximal inhibition of 62 and 37 %) while E 10 did not inhibit at all the enzyme at this concentration (Fig. 3). When decreasing the concentrations, the inhibitory effect was almost completely lost at 0.01 mg/mL (see Additional file 3). Clearly, even at the higher concentration, the extracts were much less efficient than isofagomine.

### Fractionation of extracts and screening on α-glucosidase activity

**Fig. 4:**
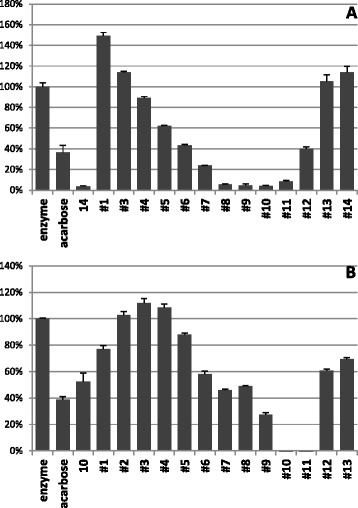
Inhibition of α-glucosidase by subfractions separated from extracts 14 (panel **a**) and 10 (panel **b**). α-Glucosidase was incubated with extracts at a concentration of 1 mg/mL. Remaining activities in the presence of extracts are expressed as a percentage of enzyme activity incubated in DMSO alone and are the mean ± SE of three separate determinations. 10 mM Acarbose was used as specific inhibitor of α-glucosidase

To go further in the identification of active compounds, the most efficient raw extract from *N. diderrichii* (E 14, Fig. 4a) and that from *S. pobeguinii* (E 10, Fig. 4b) were submitted to preparative HPLC in order to evidence the most efficient fractions by biological evaluation (see Additional files 4 and 5). The subfractions were tested on α-glucosidase activity and compared to the raw extracts 10 and 14. The head hydrophilic fractions as well as the queue lipophilic fractions displayed a moderate inhibitory potency at a concentration of 1 mg/mL, whereas fractions 10.10 and 10.11 as well as 14.8-14.11 showed the highest activity against α-glucosidase (Fig. 4).

**Fig. 5:**
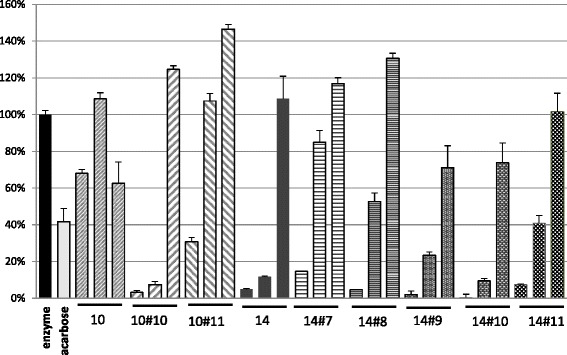
Inhibition of α-glucosidase by extracts 10 and 14 and by the most active subfractions tested at 1, 0.1 and 0.01 mg/mL (from *left* to *right*). Remaining activities in the presence of extracts are expressed as a percentage of enzyme activity incubated in DMSO alone and are the mean ± SE of three separate determinations. 10 mM Acarbose was used as specific inhibitor of α-glucosidase

These results have been corroborated by testing the active fractions at concentrations 1.0, 0.1 and 0.01 mg/mL; extract 14 remained more efficient than extract 10. The most active fractions were F 10.10, F 14.9 and F14.10, which still show very potent inhibitory effect at 0.1 mg/mL (78-93 % inhibition) (Fig. 5).

## Conclusion

The present study clearly indicates that compounds present in extracts of *N. diderrichii* (E 14 and E 15) and *S. pobeguinii* (E 10) are potent and selective inhibitors of α-glucosidase. A comparison with the results obtained with acarbose, which is used as oral medication to treat type 2 diabetes, indicates that the most active extract, obtained from *N. diderrichii* leaves, is more than 60-fold more active than the commercial antidiabetic drug. In addition, at the concentration inducing the highest effect on α-glucosidase inhibition, no toxicity was observed on human cell line. This demonstrates the safety of these extracts and permits to allow their use.

Recently, extracts with high phenolic contents have shown a strong capacity to inhibit α-glucosidase activity [54–56] and to treat diabetic rats [57]. The same correlation is observed in our study, the most active extract (E14), obtained from *N. diderrichii* leaves, being also the richest in phenols. Therefore, we can postulate that the antidiabetic activity of *N. diderrichii* and *S. pobeguinii* extracts might be related to their phenolic compounds content, even if we cannot exclude the participation of other classes of compounds.

Finally, our results validate the folk medicinal use of *S. pobeguinii* and *N. diderrichii* for their hypoglycemiant effect. Since these plants are widely available in Gabon, they could be a source of cheap and effective medication for persons with high blood glucose levels.

## Additional files

Additional file 1:
**HPTLC of extracts 10, 14 and 15 using DPPH derivatization reagent.** The DPPH test shows some very prominent antioxidant zones. (PDF 17 kb) Additional file 2:
**Diagram showing inhibition of α-glucosidase by extracts 14 and 15 at three concentrations.** α-Glucosidase was incubated with extracts at a concentration of 1, 0.1 and 0.01 mg/mL. Remaining activities in the presence of extracts are expressed as a percentage of enzyme activity incubated in DMSO alone and are the mean ± SE of three separate determinations. 10 mM Acarbose was used as specific inhibitor of α-glucosidase. (PDF 30 kb) Additional file 3:
**Diagram showing inhibition of β-glucosidase by extracts 14 and 15 at three concentrations.** β-Glucosidase was incubated with extracts at a concentration of 1, 0.1 and 0.01 mg/mL. Remaining activities in the presence of extracts are expressed as a percentage of enzyme activity incubated in DMSO alone and are the mean ± SE of three separate determinations. 1 μM Isofagomine was used as specific inhibitor of β-glucosidase. (PDF 30 kb) Additional file 4:
**Preparative HPLC fractionation curve of the raw extract from** ***N. diderrichii*** **leaves (E14).** (PDF 207 kb) Additional file 5:
**Preparative HPLC fractionation curve of the raw extract from** ***S. pobeguinii*** **bark (E10).** (PDF 189 kb)
