# Supplementary material for: Antidiabetic potential of two medicinal plants used in Gabonese folk medicine
Source: BMC Complement Altern Med. 2016 Feb 22;16:71. doi: 10.1186/s12906-016-1052-x (PMC4763413; doi:10.1186/s12906-016-1052-x)

Additional file 1

HPTLC of extracts 10, 14 and 15 using DPPH derivatization reagent.

The DPPH test shows some very prominent antioxidant zones

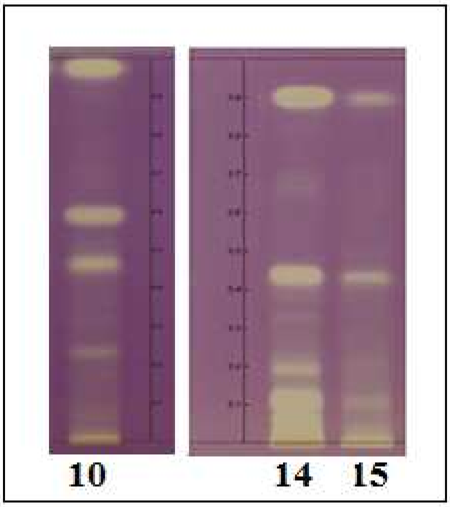

Supplement: Additional file 1: — HPTLC of extracts 10, 14 and 15 using DPPH derivatization reagent. The DPPH test shows some very prominent antioxidant zones. (PDF 17 kb) [file 12906_2016_1052_MOESM1_ESM.pdf]
