# Supplementary material for: Antidiabetic potential of two medicinal plants used in Gabonese folk medicine
Source: BMC Complement Altern Med. 2016 Feb 22;16:71. doi: 10.1186/s12906-016-1052-x (PMC4763413; doi:10.1186/s12906-016-1052-x)

Additional file 2

Diagram showing inhibition of α-glucosidase by extracts 14 and 15 at three concentrations.

α-Glucosidase was incubated with extracts at a concentration of 1, 0.1 and 0.01 mg/mL. Results are expressed as the percentage of activity of enzyme incubated with DMSO alone and are the mean ± SE of three separate determinations. 10 mM Acarbose was used as specific inhibitor of α-glucosidase.

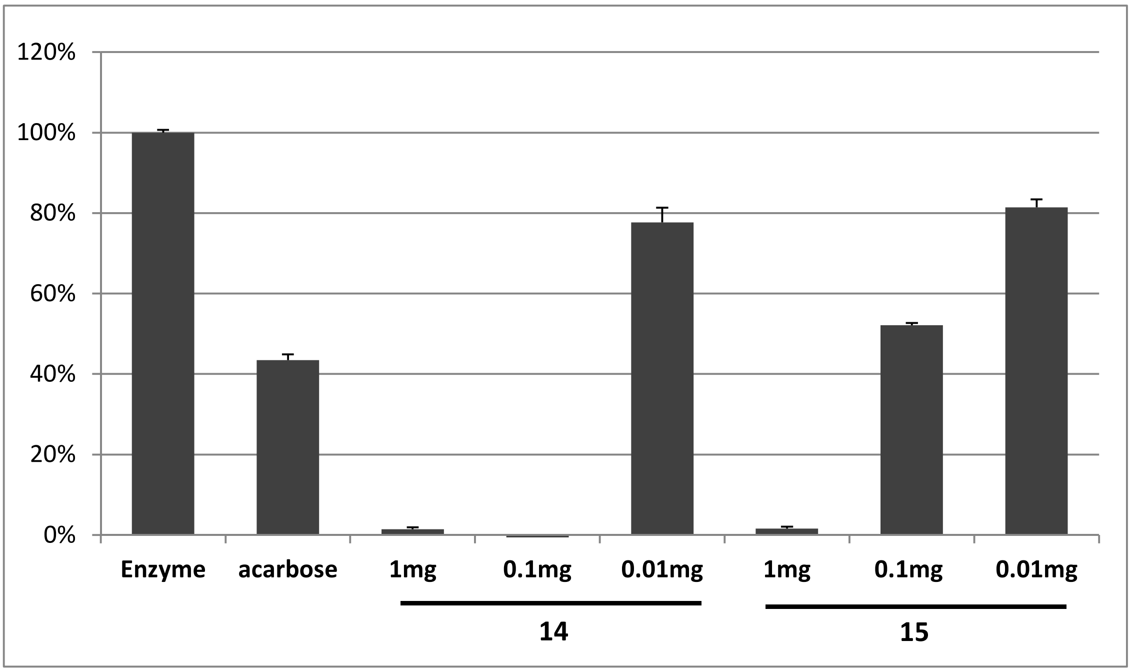

Supplement: Additional file 2: — Diagram showing inhibition of α-glucosidase by extracts 14 and 15 at three concentrations. α-Glucosidase was incubated with extracts at a concentration of 1, 0.1 and 0.01 mg/mL. Remaining activities in the presence of extracts are expressed as a percentage of enzyme activity incubated in DMSO alone and are the mean ± SE of three separate determinations. 10 mM Acarbose was used as specific inhibitor of α-glucosidase. (PDF 30 kb) [file 12906_2016_1052_MOESM2_ESM.pdf]
