# Supplementary material for: Antidiabetic potential of two medicinal plants used in Gabonese folk medicine
Source: BMC Complement Altern Med. 2016 Feb 22;16:71. doi: 10.1186/s12906-016-1052-x (PMC4763413; doi:10.1186/s12906-016-1052-x)

Additional file 3

Diagram showing inhibition of β-glucosidase by extracts 14 and 15 at three concentrations

β-Glucosidase was incubated with extracts at a concentration of 1, 0.1 and 0.01 mg/mL. Results are expressed as the percentage of activity of enzyme incubated with DMSO alone and are the mean ± SE of three separate determinations. 1 µM Isofagomine was used as specific inhibitor of β-glucosidase.

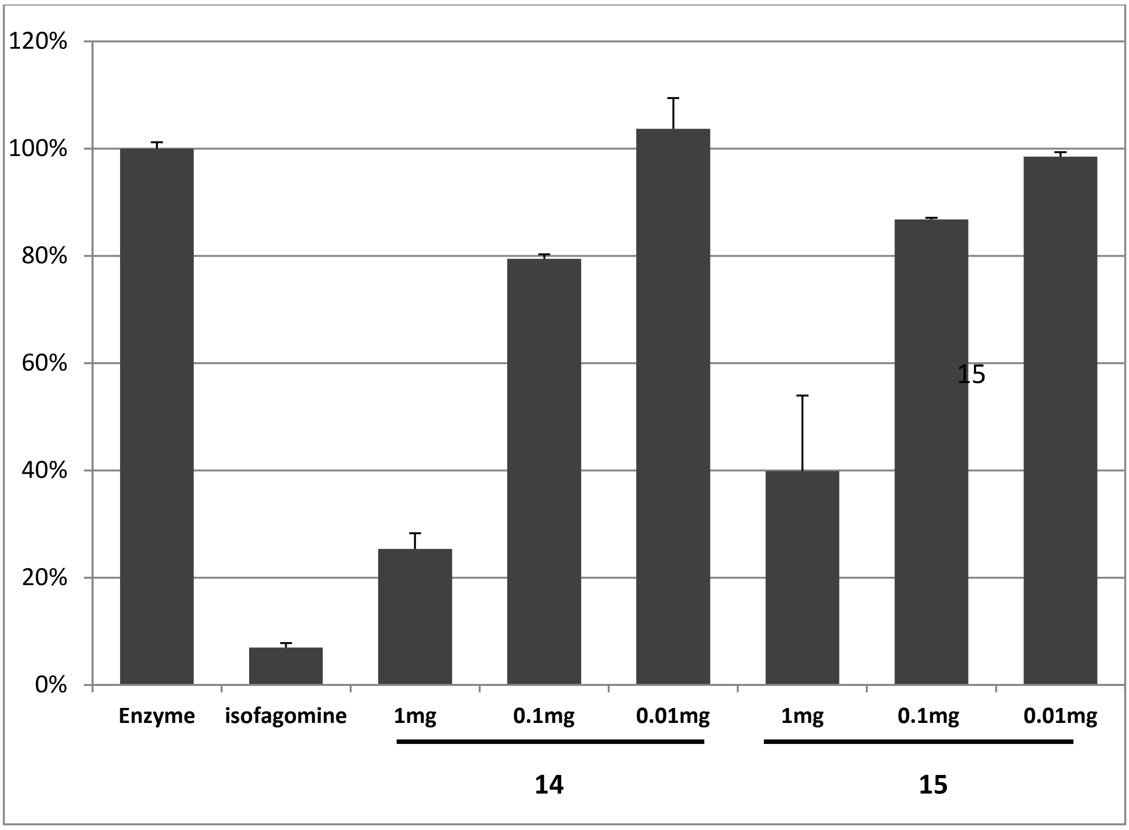

Supplement: Additional file 3: — Diagram showing inhibition of β-glucosidase by extracts 14 and 15 at three concentrations. β-Glucosidase was incubated with extracts at a concentration of 1, 0.1 and 0.01 mg/mL. Remaining activities in the presence of extracts are expressed as a percentage of enzyme activity incubated in DMSO alone and are the mean ± SE of three separate determinations. 1 μM Isofagomine was used as specific inhibitor of β-glucosidase. (PDF 30 kb) [file 12906_2016_1052_MOESM3_ESM.pdf]
