# Supplementary material for: Antidiabetic potential of two medicinal plants used in Gabonese folk medicine
Source: BMC Complement Altern Med. 2016 Feb 22;16:71. doi: 10.1186/s12906-016-1052-x (PMC4763413; doi:10.1186/s12906-016-1052-x)

Additional file 5

Preparative HPLC fractionation curve of the raw extract from *S. pobeguinii* bark (E10)

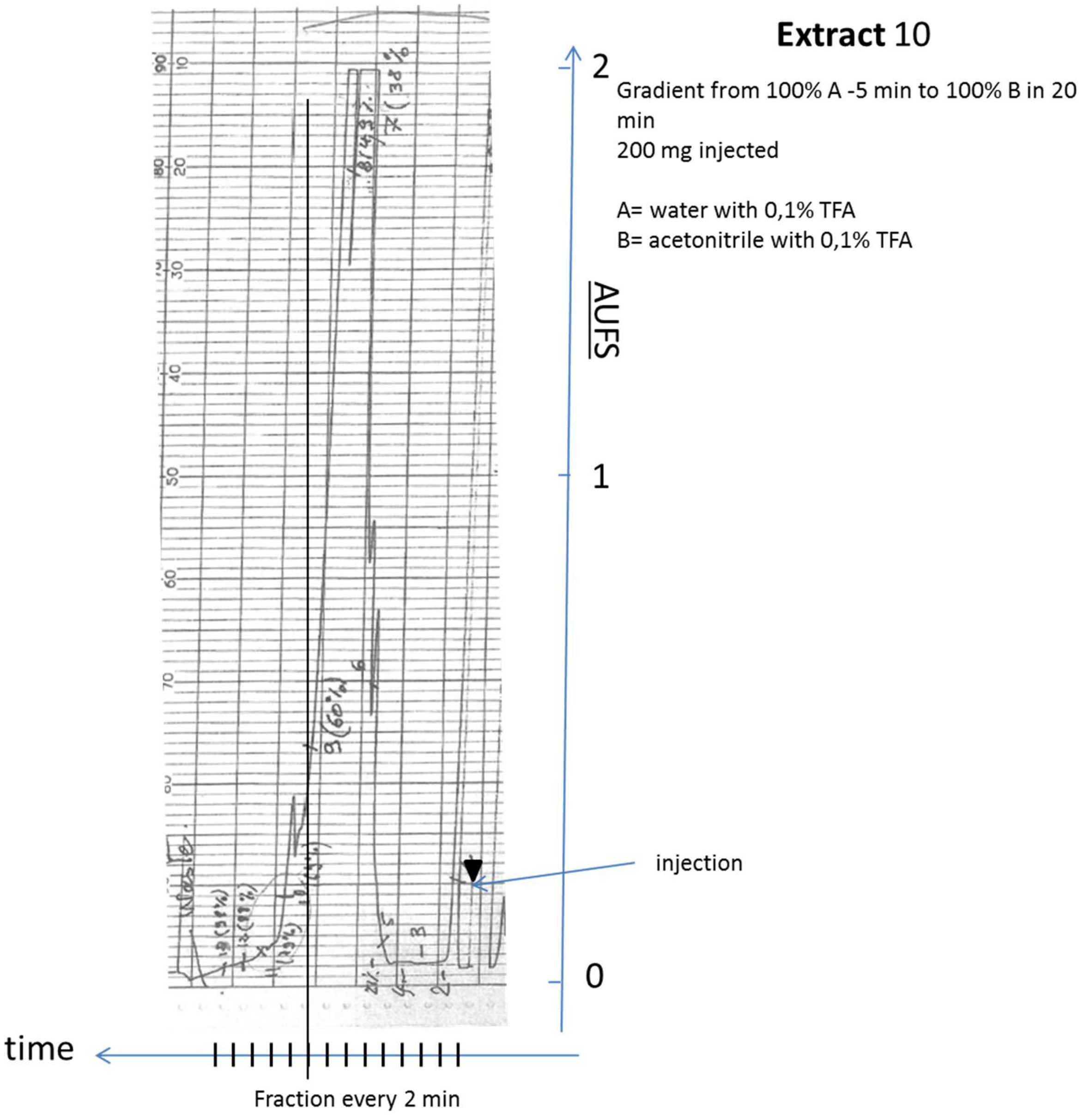

Supplement: Additional file 5: — Preparative HPLC fractionation curve of the raw extract from S. pobeguinii bark (E10). (PDF 189 kb) [file 12906_2016_1052_MOESM5_ESM.pdf]
